# Cadmium exposure and risk of prostate cancer: a meta-analysis of cohort and case-control studies among the general and occupational populations

**DOI:** 10.1038/srep25814

**Published:** 2016-05-13

**Authors:** Cheng Chen, Pengcheng Xun, Muneko Nishijo, Sue Carter, Ka He

**Affiliations:** 1 Department of Epidemiology and Biostatistics, School of Public Health–Bloomington, Indiana University, Bloomington, IN 47405, USA; 2 Department of Epidemiology and Public Health, Kanazawa Medical University, Ishikawa 920-0293, Japan; 3 Kinsey Institute, Indiana University, Bloomington, IN 47405, USA

## Abstract

We aimed to evaluate the association of cadmium exposure with the risk of prostate cancer in both the general and occupational populations. Online database searches were performed for studies of prostate cancer risk and cadmium exposure. Twelve cohort studies (5 in the general, 7 in occupational populations) and 9 case-control studies (3 in the general, 6 in occupational populations) were identified. Five/seven cohort studies in the general and occupational populations consist of 78,263/13, 434 participants with a mean follow-up of 12.1/43.0 years, respectively. Case-control studies include 334 cases/670 controls in the general population, and 1,315 cases/4,477 controls in occupational populations. Comparing the highest to the lowest category of cadmium exposure in the general population, the weighted relative risk of prostate cancer incidence and mortality among cohort studies, and the weighted odds ratio in case-control studies were 1.05 (95%CI [0.91, 1.22]), 0.83 (95%CI [0.35, 1.98]), and 1.27 (95%CI [0.58,2.78]), respectively. For occupational populations, the weighted OR in case-control studies was 1.17 (95%CI [0.85, 1.62]), and the weighted standardized mortality ratio in cohort studies was 98 (95%CI [75, 126]). Accumulated epidemiological evidence does not support the hypothesis that cadmium exposure may increase the risk of prostate cancer in either the general or occupational populations.

Prostate cancer is the leading type of cancer among men in the USA and worldwide^1^. Investigation of its risk factors has practical importance for public health efforts, since causes of prostate carcinogenesis are largely unknown^2^. Cadmium is a redundant occupational and environmental contaminant that has been suspected to induce human prostatic carcinogenesis^3^. Workers of a large variety of occupations, especially those are involved in manufacture of alloy and battery, and nonferrous metal smelting and refining^4^, are exposed to high level of cadmium through inhalation of dust and fumes, and incidental ingestion of dust from contaminated hands, cigarettes or foods^5^. In addition, environmental exposure to cadmium among the general population has become a concern due to the extensive use of cadmium in industries and the subsequent soil, water, and air pollution^5^.

Early epidemiological studies found elevated prostate cancer mortality among cadmium exposed workers^6^, whereas later studies failed to confirm this positive association^7^,^8^,^9^. While occupational studies showed inconsistent results, the risk of prostate cancer among the general population who has relatively lower intensity of cadmium exposure is unclear. An increased risk of prostate cancer with higher environmental cadmium exposure was observed among the general population in some studies^10^,^11^, but not in others^12^,^13^,^14^,^15^,^16^. Two systematic reviews in early 2000s both concluded that epidemiological studies to date had not yielded sufficient evidence supporting the hazard of cadmium exposure to human prostate^17^,^18^, but, no meta-analysis has been conducted to quantitatively examine the risk of prostate cancer in relation to cadmium exposure. Thus, we aimed to quantitatively assess the overall association between cadmium exposure and the risk of prostate cancer in both the general and occupational populations and to explore the dose-response relationship with updated literature.

## Methods

### Study selection

This meta-analysis was carried out based on the criteria of Preferred Reporting Items for Systematic reviews and Meta-analyses (PRISMA). The relevant observational studies published in English, which investigated the association between cadmium exposure and the risk of prostate cancer, were identified by searching PubMed database from inception to June 2015 using the terms “cadmium” (MeSH terms) cross-referenced to “prostate cancer” (MeSH terms). Google Scholar and reference lists of narrative and systematic reviews and the relevant articles were searched for additional citations.

Two reviewers (C.C. and P.X.) independently reviewed all relevant articles. Disagreements were resolved by group discussion. To be considered for inclusion, studies had to meet the following criteria: a) cohort or case-control studies; and b) hazard ratio (HR), relative risk (RR), standardized mortality ratio (SMR), or odds ratio (OR) with corresponding 95% confidence intervals (95% CIs) of prostate cancer in relation to cadmium exposure were reported, or such information could be derived from the published results. We also included unpublished *de novo* results provided by *Qian et al.*^19^.

**Figure 1:**
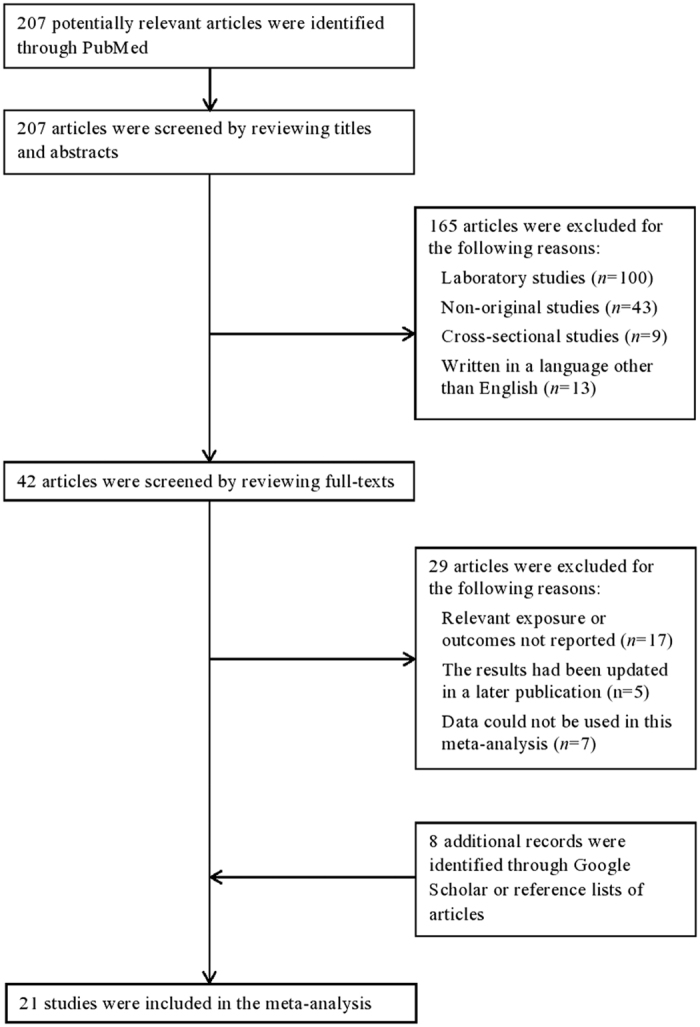
Process of study selection.

Fig.1 shows the detailed study selection process. We excluded articles if they were laboratory studies (*n* = 100), non-original studies (reviews or letter-to-editors) (*n* = 43), or cross-sectional studies (*n* = 9); if they were not published in English language (*n* = 13); if the exposure or the outcome was not cadmium or prostate cancer (*n* = 17); if the results had been updated in a later publication that was included (*n* = 5); or if the available data could not be combined with other studies and requests for *de novo* results were not successful (*n* = 7). Eight additional articles were identified through Google Scholar or reference lists of articles. In sum, 21 studies (5 cohort and 3 case-control studies in the general population, and 7 cohort and 6 case-control studies in occupational populations) met the criteria and were included in this meta-analysis.

### Data extraction

Two of the co-authors (C.C. and P.X.) independently assessed each study and extracted the relevant information. Discrepancies were resolved by group discussion. We recorded the following information (when available): the first author’s last name, year of publication, region of study, number of participants and events (or number of cases and controls), age of participants, follow-up years (for cohort studies), outcome confirmation (or case identification) method, and measurements of the association of interest. In addition, for cohort studies in the general population, and case-control studies in the general and occupational populations, we collected information on exposure assessment method, categories of exposure, and adjusted covariates in the final model; for occupational cohort studies, information on exposed population, reference population, and observed/expected cases was extracted. In particular, HR, RR, and OR with 95% CIs for all cadmium exposure categories versus the lowest exposure group^10^,^11^,^12^,^13^,^14^,^15^,^16^,^19^,^20^,^21^,^22^,^23^,^24^,^25^, and SMR (*100) with 95% CIs for cadmium exposed workers^7^,^8^,^9^,^26^,^27^,^28^,^29^ were collected.

### Statistical analysis

The association between cadmium exposure and the risk of prostate cancer was expressed as weighted RR (HR was considered as RR) among cohort studies in the general population and OR in case-control studies as comparing the highest to the lowest category of cadmium exposure. Both RR and OR were transformed to their natural logarithms (ln) and the corresponding 95% CIs were used to calculate their standard errors.

Dose-response relationship between cadmium exposure and prostate cancer risk was estimated based on available categorical RRs using meta-regression method^30^. The overall dose-response relationship was examined in two cohort studies^10^,^13^ in the general population.

The association of interest among occupational cohort studies was expressed as weighted SMR (*100). SMR was transformed to its natural logarithm (ln) and the standard error was calculated by using Poisson probability distribution method^31^.

Random-effects model was used for all analyses because the primary studies were conducted among different populations and heterogeneity is not negligible. Sensitivity analyses were performed to detect the influence of any single study on the overall association. Statistical heterogeneity was explored and quantified by using *I*^*2*^ statistic along with Cochran’s Q test. Publication bias were only assessed when the number of studies ≥6 by Egger’s regression asymmetry test^32^. A two-sided P value ≤ 0.05 was considered statistically significant. All analyses were performed with STATA software (Version 13.1, STATA Corporation LP, College Station, TX).

## Results

### Study characteristics

Twenty-one studies were identified in this meta-analysis. Five^10^,^12^,^13^,^14^,^19^/seven^7^,^8^,^9^,^26^,^27^,^28^,^29^ cohort studies in the general/occupational populations consist of 78,263/13,434 participants (4,731/83 events) with a mean follow-up of 12.1/43.0 years (Supplemental Tables 1 and 2). Three^11^,^15^,^16^/six^20^,^21^,^22^,^23^,^24^,^25^ case-control studies in the general/occupational populations include 334/1,315 cases and 670/4,477 controls (Supplemental Tables 3 and 4).

Among the 5 cohort studies in the general population, multivariable adjusted RRs of prostate cancer incidence by tertiles of dietary cadmium intake were reported in 2 studies^10^,^13^; multivariable adjusted HRs or RRs of prostate cancer mortality based on tertiles or quartiles of urinary cadmium concentrations were estimated in the other 3 studies^12^,^14^,^19^. Seven cohort studies in occupational populations^7^,^8^,^9^,^26^,^27^,^28^,^29^ assessed SMRs (*100) of cadmium exposed workers by comparing observed to expected number of death estimated from the corresponding national or local populations. Three case-control studies in the general population and 6 occupational case-control studies reported multivariate-adjusted ORs of prostate cancer risk in relation to quartiles or quintiles of toenail cadmium concentrations^11^,^16^, or quartiles of dietary cadmium intake^15^, or multiple author-defined levels of cadmium exposure^20^,^21^,^22^,^23^,^24^,^25^.

### Meta-analysis

**Figure 2:**
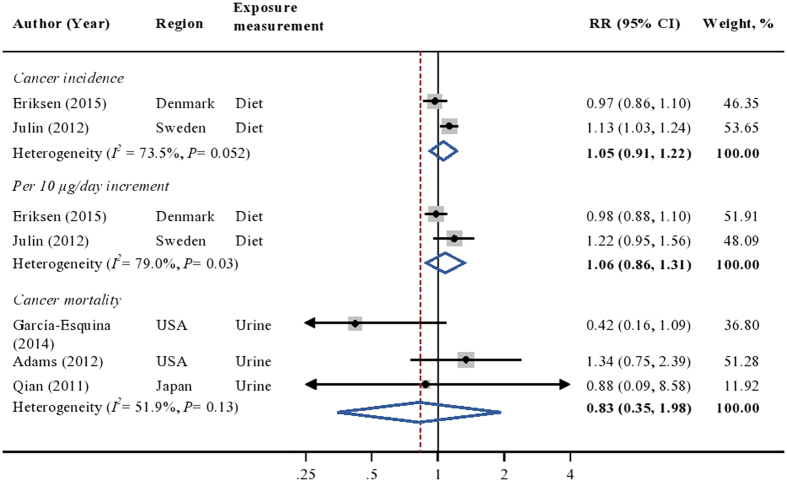
Multivariable adjusted RRs and 95% CIs of prostate cancer by cadmium exposure in 5 cohort studies among the general population. The summary estimates were obtained using a random-effects model. The dots indicate the adjusted RRs by comparing the highest to the lowest level of cadmium exposure or with one unit increment in cadmium exposure. The size of the shaded square is proportional to the percent weight of each study. The horizontal lines represent 95% CIs. The diamond data markers indicate the summary RRs. CI: confidence interval; RR: relative risk.

Comparing the highest to the lowest cadmium exposure category, the weighted RR among cohort studies in the general population did not suggest an association between cadmium exposure and prostate cancer incidence (RR = 1.05; 95%CI [0.91, 1.22]; Fig. 2) or mortality (RR = 0.83; 95%CI [0.35, 1.98]; Fig. 2). Also, no linear relation was observed between cadmium exposure and prostate cancer incidence (RR = 1.06; 95%CI [0.86, 1.31]; Fig. 2), though high heterogeneity was found (*I*^*2*^ = 79.0%, *P* = 0.03). There was insufficient data to detect possible linear relation among studies on prostate cancer mortality. Sensitivity analyses showed no single study appreciably changed the results.

**Figure 3:**
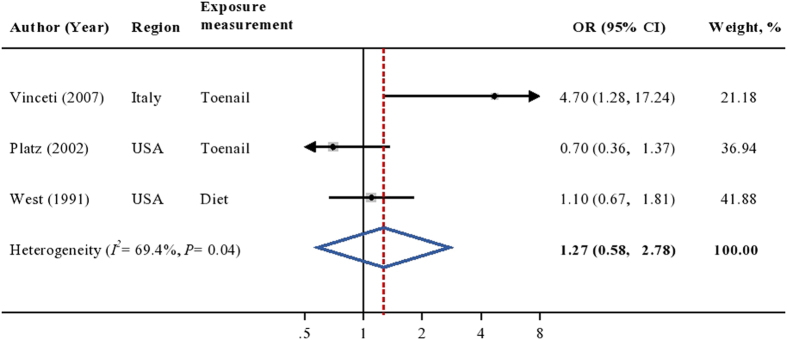
Multivariable adjusted OR and 95% CI of prostate cancer risk by cadmium exposure in 3 case-control studies in the general population. The summary estimate was obtained using a random-effects model. The dots indicate the adjusted ORs by comparing the highest to the lowest level of cadmium exposure. The size of the shaded square is proportional to the percent weight of each study. The horizontal lines represent 95% CIs. The diamond data marker indicates the summary OR. CI: confidence interval; OR: odds ratio.

**Figure 4:**
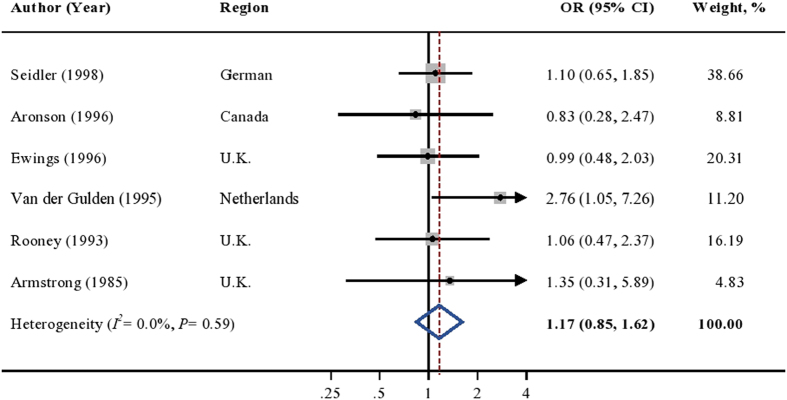
Multivariable adjusted OR and 95% CI of prostate cancer risk by cadmium exposure in 6 occupational case-control studies. The summary estimate was obtained using a random-effects model. The dots indicate the adjusted ORs by comparing the highest to the lowest level of cadmium exposure. The size of the shaded square is proportional to the percent weight of each study. The horizontal lines represent 95% CIs. The diamond data marker indicates the summary OR. CI: confidence interval; OR: odds ratio.

Among the case-control studies conducted in the general population, the weighted OR did not reveal an association between cadmium exposure and the risk of prostate cancer (OR = 1.27; 95%CI [0.58, 2.78]; Fig. 3), though significant heterogeneity was observed in the analysis (*I*^*2*^ = 69.4%, *P* = 0.04). Sensitivity analyses found no any single study affected the overall estimate considerably. Similar results were found in the occupational case-control studies (weighted OR = 1.17; 95%CI [0.85, 1.62]; Fig. 4). The pooled association persisted when omitting any single study at each time. Evidence on heterogeneity (*I*^*2*^ = 0.0%, *P* = 0.59) and publication bias (*P* = 0.61) were not found.

**Figure 5:**
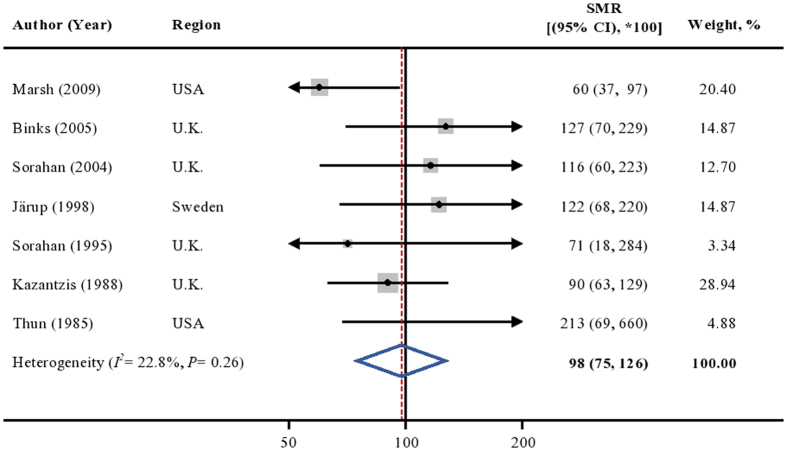
SMR (*100) and 95% CI of prostate cancer in relation to cadmium exposure in 7 occupational cohort studies. The summary estimate was obtained using a random-effects model. The dots indicate the SMRs (*100). The size of the shaded square is proportional to the percent weight of each study. The horizontal lines represent 95% CIs. The diamond data marker indicates the summary SMR. CI: confidence interval; SMR: standardized mortality ratio.

In addition, the weighted SMR (*100) among occupational cohort studies did not indicate any significant association (SMR = 98; 95%CI [75, 126]; Fig. 5). The result was not materially influenced by any single study. Heterogeneity (*I*^*2*^ = 22.8%, *P* = 0.26) and publication bias (*P* = 0.35) were not detected.

## Discussion

The accumulated literature did not provide solid evidence supporting an association between cadmium exposure and the risk of prostate cancer in either the general or occupational populations. Although our findings are consistent with the results in two previous systematic reviews^17^,^18^ published 10 years ago, most studies in these two systematic reviews have been updated and 10 additional studies are included in the present meta-analysis.

Some limitations in this meta-analysis need to be considered. First, although our study has combined the most comprehensive and updated findings in literature, primary studies in the general population are limited, especially those used the same exposure assessment method and had similar ranges of cadmium exposure, which is probably the source of high heterogeneity when combining case-control studies and when estimating the dose-response relationship among cohort studies. However, the cadmium levels estimated from dietary surveys and toenail samples should be generally parallel to each other and will enable us to compute the relative risk of prostate cancer by ranking participants based on cadmium exposure levels. Meanwhile, according to previous literature^10^,^11^,^12^, we reasonably assumed a linear relationship between cadmium exposure and prostate cancer risk and estimated the pooled RR/OR based on this assumption. Of note, the pooled association was not changed when omitting any single study each time in the analysis. Second, case-controls studies included in this meta-analysis are composed of hospital-11,20,21,23 and population-based^15^,^16^,^22^,^24^,^25^ studies, which may increase the heterogeneity, even though sensitivity analyses suggested no single study appreciably influenced the results. Third, a potential publication bias resulting from the exclusion of articles published in a language other than English or any unpublished result could not be completely excluded, though Egger’s regression asymmetry test did not suggest publication bias in the present meta-analysis when pooling studies ≥6. In addition, we have reviewed the English abstract (if available) of the excluded studies published in other languages. None of them met the inclusion criteria.

In contrast to epidemiological studies, numerous experimental studies have confirmed the carcinogenic property of cadmium on human prostate *in vivo* and *in vitro*^33^. One explanation for the conflicting evidence may be the imprecision in the assessment of cadmium exposure. Occupational studies relying on the job history and studies in the general population without objective measurement on cadmium exposure may increase the probability of recall bias, information bias and/or misclassification. In addition, occupational studies did not adjust for concomitant exposure to other carcinogens may make it difficult to determine the hazard of cadmium exposure alone. Similarly, the null association observed in studies in the general population may be confounded by potential protective factors of prostate cancer, such as high vegetable and fruit intake^34^. Furthermore, the association between cadmium exposure and prostate cancer risk may be attenuated by the competing risk of lung cancer, which is the most prevalent and deadly type of cancer. Participants are highly likely to die from lung cancer before they develop prostate cancer^1^.

In summary, this updated meta-analysis does not generate solid evidence supporting a positive association between cadmium exposure and the risk of prostate cancer in either the general or occupational populations. Extended follow-up of existing cohort studies, particularly in the general population, along with further epidemiological research with objective assessment of cadmium exposure, are needed to accurately determine the role of cadmium in the development of human prostate cancer. Of note, prostatic cancer can be found at early stage using serum PSA measurement and curable by medical treatments in recent years, which suggests that incidence studies, in addition to mortality studies, are also necessary to conclude the prostatic cancer risk in relation to cadmium exposure. The null association observed in the present meta-analysis should not change any ongoing public health efforts to eliminate cadmium exposure of industrial workers and cadmium contamination in environment, which may have detrimental influence on human health, especially at high exposure levels, based on the existing literature.

## Additional Information

**How to cite this article**: Chen, C. *et al.* Cadmium exposure and risk of prostate cancer: a meta-analysis of cohort and case-control studies among the general and occupational populations. *Sci. Rep.* **6**, 25814; doi: 10.1038/srep25814 (2016).

## Supplementary Material

Supplementary Tables
