# Supplementary material for: Cadmium exposure and risk of prostate cancer: a meta-analysis of cohort and case-control studies among the general and occupational populations
Source: Sci Rep. 2016 May 13;6:25814. doi: 10.1038/srep25814 (PMC4865877; doi:10.1038/srep25814)
Supplement: Supplementary Tables [file srep25814-s1.doc]

**Supplemental information**

**Cadmium exposure and risk of prostate cancer: a meta-analysis of cohort and case-control studies among the general and occupational populations**

Cheng Chen, Pengcheng Xun, Muneko Nishijo, Sue Carter, Ka He

| **Table 1** Characteristics of the 5 cohort studies in the general population | | | | | | | | | |
| --- | --- | --- | --- | --- | --- | --- | --- | --- | --- |
| **Author (year)** | **Region** | **No. of participants (events)** | **Age^a^,**  **y** | **Follow-up^b^, y** | **Exposure assessment** | **Outcome confirmation** | **Exposure**  **categories** | **Risk**  **estimate** | **Adjusted covariates** |
| *Cancer incidence* | | | | | | | | | |
| Eriksen  (2015) | Denmark | 26,778 (1,567) | 50-65 | 13 | Dietary Cd by FFQ | Danish Cancer Registry,  ICD-10  (code C61) | Dietary Cd intake  (tertiles, μg/day):  <14;  14-18;  >18. | RR (95% CI):  1.00 (referent);  0.96 (0.85-1.08);  0.97 (0.86-1.10).  Per 10 μg/day increment:  0.98 (0.88-1.10). | Education level, smoking status, BMI, waist-to-hip ratio, and physical activity. |
| Julin  (2012) | Sweden | 41,089 (3,085) | 45-79 | 10.8 | Dietary Cd by FFQ | National Cancer Registry | Dietary Cd intake  (tertiles, μg/day):  <17;  17-20;  >20. | RR (95% CI):  1.00 (referent);  1.11 (1.01-1.21);  1.13 (1.03-1.24).  Per 10 μg/day increment:  1.22 (0.95-1.56). | Age, family history of prostate cancer, years of education, BMI, waist circumference, physical activity, smoking status, total energy intake, alcohol consumption, selenium, lycopene, and calcium intake. |

| **Table 1** continued | | | | | | | | | |
| --- | --- | --- | --- | --- | --- | --- | --- | --- | --- |
| **Author (year)** | **Region** | **No. of participants (events)** | **Age^a^,**  **y** | **Follow-up^b^, y** | **Exposure assessment** | **Outcome confirmation** | **Exposure**  **categories** | **Risk**  **estimate** | **Adjusted covariates** |
| *Cancer mortality* | | | | | | | | | |
| García-Esquina  (2014) | USA | 1,538 (16) | 45-75 | 17.2 | Urinary Cd by inductively coupled plasma mass spectrometry  (Agilent 7700x ICPMS; Agilent Technologies, Waldbronn Germany) | Death certificates and autopsy records  ICD-9 (code185) | uCd  (tertiles, μg/g Cr):  ≤0.70;  0.71-1.22;  ≥1.23. | HR (95% CI):  1.00 (referent);  1.37 (0.40-4.66);  0.48 (0.11-2.08).  80^th^ *vs*. 20^th^:  0.42 (0.16-1.08). | Gender, age, smoking status, pack-years of smoking, and BMI. |
| Adams  (2012) | USA | 7,455 (54) | ≥17 | 13.4 | Urinary Cd by Perkin-Elmer Model 3030 atomic absorption  spectrometry with Zeeman background correction  Urinary Cr by the Jaffe method with an ASTRA analyzer | Death certificates and the National Death Index  ICD-9 to ICD-10 | uCd  (quartiles, μg/g Cr):  ≤0.153;  0.154-0.297;  0.298-0.579;  ≥0.580. | HR (95% CI)  4^th^ quartile vs  1-3^rd^ quartiles:  1.34 (0.75-2.38). | Age, smoking history, BMI, education, and race. |
| Qian^c^  (2011) | Japan | 1,403 (9) | ≥50 | 22 | Urinary Cd by atomic absorption spectrometry  Urinary Cr by the Jaffe method | Death certificates,  ICD-9 | uCd  (quartiles, μg/g Cr):  <3.0;  3.0-4.9;  5.0-9.9;  ≥10.0. | RR (95% CI):  1.00(referent);  1.22 (0.27-0.55);  0.30 (0.03-2.92);  0.88 (0.09-8.56). | Age. |
| ^a^ Range of age was reported.  ^b^ The mean or median years of follow-up were reported.  ^c^ The authors provided de novo results, which were not reported in their primary article, to this meta-analysis.  BMI, body mass index; Cd, cadmium; FFQ, food frequency questionnaire; HR, hazard ratio; ICD, International Classification of Diseases; RR, relative risk; uCd, urinary cadmium concentration; 95% CI, 95% confidence interval. | | | | | | | | | |

| **Table 2** Characteristics of the 7 cohort studies in occupational populations | | | | | | | | | |
| --- | --- | --- | --- | --- | --- | --- | --- | --- | --- |
| **Author (Year)** | **Region** | **No. of participants** | **Age^a^,**  **y** | **Follow-up^b^, y** | **Outcome confirmation** | **Exposed**  **population** | **Reference population^c^** | **Observed**  **/expected**  **cases** | **Risk Estimate**  **[SMR (95% CI), *100]** |
| Marsh  (2009) | USA | 2,422 | -- | 51 | Death certificate ICD-9 (code185) | Coper mining, mining, and smelter facility workers | General U.S. population | 17/28.6 | 60 (35-95) |
| Binks  (2005) | U.K. | 1,462 | -- | 34 | Death certificate | Tin smelter workers | General England and Wales population | 11/8.68 | 127 (63-227) |
| Sorahan  (2004) | U.K. | 926 | 14-84 | 53 | Death certificate  ICD-8 (code 162); ICD-9 (code 162) | Nickel-cadmium battery workers | General England and Wales population | 9/7.5 | 116 (53-221) |
| Järup  (1998) | Sweden | 717 | -- | 41 | National Swedish cause of death registry  Swedish cancer registry  ICD-8 | Nickel-cadmium battery workers | Kalmar county population | 11/9.0 | 122 (61.1-219) |
| Sorahan  (1995) | U.K. | 347 | ≥25 | 50 | Death certificate ICD-8 (code185) | Copper cadmium alloy workers | General England and Wales population | 2/2.83 | 71 (9-255) |
| Kazantzis (1988) | U.K. | 6,958 | -- | 41 | Death certificate ICD-8 (code185) | Primary production, copper-cadmium alloys, silver-cadmium alloys, pigments and oxide, and stabilizers | General England and Wales population | 30/33.2 | 90 (61-129) |
| Thun  (1985) | USA | 602 | -- | 38 | Death certificate ICD-7 | Cadmium production workers | General U.S. population | 3/1.41 | 213 (44-622) |
| ^a^ The range of age was reported.  ^b^ The mean years were reported.  ^c^ Reference populations were used to calculate SMR.  ICD, International Classification of Diseases; SMR, standardized mortality ratio; 95% CI, 95% confidence interval; --, not available. | | | | | | | | | |

| **Table 3** Characteristics of the 3 case-control studies in the general population | | | | | | | | | |
| --- | --- | --- | --- | --- | --- | --- | --- | --- | --- |
| **Author (year)** | **Region** | **Cases,**  **n** | **Controls,**  **n** | **Age^a^,**  **y** | **Exposure assessment** | **Case confirmation** | **Exposure categories** | **Risk**  **estimate** | **Adjusted covariates** |
| Vinceti  (2007)^b^ | Italy | 40 | 58 | 43-83 | Toenail Cd assessed by Zeeman-effect corrected graphite-furnace atomic absorption spectrometer | Hospital record | Toenail Cd  (quartiles, μg/g):  <0.0073;  0.0073-0.0145;  0.0145-0.0306;  ≥0.0306. | OR (95% CI):  1.00 (referent);  0.5 (0.1-2.5);  1.3 (0.3-4.9);  4.7 (1.3-17.5). | Matched on gender and age (±5 years). |
| Platz  (2002)^c^ | USA | 115 | 227 | 66.0±7.6 | Toenail Cd assessed by Zeeman-effect corrected graphite-furnace atomic absorption spectrometer | Hospital record | Median toenail Cd adjusted for toenail weight  (quintiles, ppb):  10.8;  28.7;  54.5;  104.4;  310.8. | OR (95% CI):  1.00 (referent);  0.56 (0.28-1.13);  0.46 (0.22-0.95);  0.74 (0.38-1.44);  0.70 (0.36-1.37). | Matched on age, race, date of blood collection, and size of toenail clipping. |
| West  (1991)^c^ | USA | 179 | 385 | 45-67 | Food Frequency Questionnaire along with food composition data bank | Utah cancer registry, SEER program | Dietary Cd  (quartiles, μg/day):  <36;  36-48;  49-61;  >61. | OR (95% CI):  1.0 (referent);  1.4 (0.8-2.3);  0.8 (0.5-1.4);  1.1 (0.7-1.9). | NA |
| ^a^The mean±SD or range of age was reported.  ^b^ Hospital-based case-control studies.  ^c^ Population-based case-control studies.  Cd, cadmium; NA, no adjustment; OR, odds ratio; SEER program, the Surveillance, Epidemiology, and End Results program; 95% CI, 95% confidence interval. | | | | | | | | | |

| **Table 4** Characteristics of the 6 case-control studies in occupational populations | | | | | | | | | | | |
| --- | --- | --- | --- | --- | --- | --- | --- | --- | --- | --- | --- |
| **Author (year)** | **Region** | **Cases, n** | **Controls, n** | **Age^a^,**  **y** | **Exposure**  **assessment** | **Case confirmation** | **Exposure categories** | **Risk**  **estimate** | **Adjusted**  **covariates** |  | |
| Seidler  (1998)^b^ | German | 187 | 204 | 70.4  ± 8.6 | Job-exposure matrix based on the presence, frequency, and intensity of exposure using self-administrable socio-demographic and job history questionnaire | Hospital record | Cadmium  (dose-years):  0;  0-25. | OR (95% CI):  1.0 (referent);  1.1 (0.6-1.7). | Age, smoking, and region. |  | |
| Aronson (1996)^c^ | Canada | 449 | 2,083 | 35-70 | Expert translated socio-demographic and job history questionnaire based on the presence, frequency, and intensity of exposure | Hospital record | Unexposed;  Non-substantial exposed;  Substantial exposed. | OR (95% CI):  1.00 (referent);  0.83 (0.28-2.48);  --. | Age, ethnicity, SES, Quetelet index, status of respondent, and exposure to metallic dust, soot, aliphatic aldehydes, formaldehyde, other pyrolysis fumes, diesel dusts, lubricating oils and greases, alkanes, polyaromatic hydrocarbons, benzopyrene |  | |
| Ewings  (1996)^b^ | U.K. | 159 | 325 | -- | Cd exposure related to occupational activities and hobbies was assessed through interviews | Hospital record | Never exposed to cadmium through occupations or hobbies;  Ever exposed. | OR (95% CI):  1.00 (referent);  0.99 (0.48-2.02). | Matched on age (±5 years) | |  |

| **Table 4** continued | | | | | | | | | |
| --- | --- | --- | --- | --- | --- | --- | --- | --- | --- |
| **Author (year)** | **Region** | **Cases, n** | **Controls, n** | **Age^a^,**  **y** | **Exposure assessment** | **Case confirmation** | **Exposure categories** | **Risk**  **estimate** | **Adjusted**  **covariates** |
| Van der Gulden (1995)^b^ | Netherlands | 345 | 1,346 | 45-91 | Validated questionnaire of job history, occupational exposures and socio-demographic information | Cancer registry | Unexposed;  “Sometimes” or “frequently“  exposed ;  “Frequently” exposed. | OR (95% CI):  1.00 (referent);  1.10 (0.64-1.89);  2.76 (1.05-7.27). | Age. |
| Rooney  (1993)^c^ | U.K. | 136 | 404 | 48.4  ± 11.0 | Experts estimated occupational exposures based on detailed job histories | Cancer registry or hospital record | Never worked in a place potentially contaminated with cadmium;  Ever worked. | OR (95% CI):  1.00 (referent);  1.06 (0.46-2.30). | Matched on year of starting work at the authority, age at starting work, last place of work, ever having been monitored for exposure to ionizing radiation, and survival for at least as long as the interval between the start of employment and the date of cancer registration or death of corresponding case. |
| Armstrong (1985)^c^ | U.K. | 39 | 115 | -- | Experts estimated occupational exposures based on detailed job histories | Death certificate | Always low;  Ever medium;  Ever high. | OR (95% CI):  1.00 (referent);  1.55 (0.49-4.93);  1.35 (0.31-5.91). | Matched on plant, age, and date of birth when possible |
| ^a^ The mean±SD or range of age was reported.  ^b^ Hospital-based case-control studies.  ^c^ Population-based case-control studies.  OR, odds ratio; SES, socioeconomic status; 95% CI, 95% confidence interval; --, not available. | | | | | | | | | |
